# Circulating MicroRNAs in Extracellular Vesicles as Potential Biomarkers of Alcohol-Induced Neuroinflammation in Adolescence: Gender Differences

**DOI:** 10.3390/ijms21186730

**Published:** 2020-09-14

**Authors:** Francesc Ibáñez, Juan R. Ureña-Peralta, Pilar Costa-Alba, Jorge-Luis Torres, Francisco-Javier Laso, Miguel Marcos, Consuelo Guerri, María Pascual

**Affiliations:** 1 Department of Molecular and Cellular Pathology of Alcohol, Príncipe Felipe Research Center, 46012 Valencia, Spain; fibanez@cipf.es (F.I.); jurena@cipf.es (J.R.U.-P.); guerri@cipf.es (C.G.); 2 Emergency Department, University Hospital of Salamanca-IBSAL, 37007 Salamanca, Spain; pcosta@saludcastillayleon.es; 3 Department of Internal Medicine, University Hospital of Salamanca, University of Salamanca, Institute of Biomedical Research of Salamanca (IBSAL), 37007 Salamanca, Spain; jltorrest@saludcastillayleon.es (J.-L.T.); laso@usal.es (F.-J.L.); mmarcos@usal.es (M.M.); 4 Department of Physiology, School of Medicine and Dentistry, University of Valencia, 46010 Valencia, Spain

**Keywords:** extracellular vesicles, miRNAs, adolescent humans, adolescent mice, gender differences, ethanol, inflammation, biomarkers

## Abstract

Current studies evidence the role of miRNAs in extracellular vesicles (EVs) as key regulators of pathological processes, including neuroinflammation and neurodegeneration. As EVs can cross the blood–brain barrier, and EV miRNAs are very stable in peripheral circulation, we evaluated the potential gender differences in inflammatory-regulated miRNAs levels in human and murine plasma EVs derived from alcohol-intoxicated female and male adolescents, and whether these miRNAs could be used as biomarkers of neuroinflammation. We demonstrated that while alcohol intoxication lowers anti-inflammatory miRNA (mir-146a-5p, mir-21-5p, mir-182-5p) levels in plasma EVs from human and mice female adolescents, these EV miRNAs increased in males. In mice brain cortices, ethanol treatment lowers mir-146a-5p and mir-21-5p levels, while triggering a higher expression of inflammatory target genes (Traf6, Stat3, and Camk2a) in adolescent female mice. These results indicate, for the first time, that female and male adolescents differ as regards the ethanol effects associated with the inflammatory-related plasma miRNAs EVs profile, and suggest that female adolescents are more vulnerable than males to the inflammatory effects of binge alcohol drinking. These findings also support the view that circulating miRNAs in EVs could be useful biomarkers for screening ethanol-induced neuroinflammation and brain damage in adolescence.

## 1. Introduction

MiRNAs are one type of small noncoding RNAs (20–22 nt) involved in the post-transcriptional regulation of gene expression by binding to target mRNAs. Current studies indicate that these small RNAs have emerged as essential regulators of almost all cellular processes, but can also participate in several diseases, such as Parkinson’s disease and Alzheimer’s disease, among others [1,2]. Recent research has confirmed that miRNAs in blood derive primarily from exosomes or extracellular vesicles (EVs), which are small membrane vesicles (30–200 nm in diameter) that are released from cells as a result of the fusion of multivesicular bodies with the plasma membrane [3,4]. These particles contain a complex cargo of proteins, lipids, and nucleic acids [5], but they are especially abundant in miRNAs. Furthermore, compared to the miRNAs found directly in blood, exosomes-/EVs-containing miRNAs, are better protected from degradation, which makes them useful biomarkers [6]. More importantly, exosomes/EVs may play an important role as carriers of miRNAs through the blood brain barrier into the circulatory system [7], which make them good candidates for the prognosis of neurodegenerative diseases [8] or neuroinflammatory processes [9].

Adolescence is an important brain maturation period during which some brain regions undergo remodeling and functional changes [10,11]. All these brain changes might explain the adolescent brain’s vulnerability to the deleterious effects of ethanol [10,12]. We have demonstrated that ethanol is able to trigger the toll-like receptor 4 (TLR4) signaling immune response in glial cells [13,14], and that binge alcohol drinking in adolescent animals activates this receptor, which leads to astrogliosis and microgliosis, increases cytokines and inflammatory mediators, and causes neuroinflammation and brain damage [15,16]. Gender differences have also been shown in the effects of binge ethanol drinking in adolescence, as demonstrated by higher plasma levels of cytokines, and chemokines in human and murine female adolescents than male adolescents [17].

Several studies demonstrate that ethanol is able to alter the miRNA profile. For instance, we have recently shown that chronic ethanol treatment is able to alter the expression of the mir-183 cluster and mir-200a/b cerebral cortices of mice chronically treated with ethanol [18]. In addition, the role of EVs as cellular transmitters of the neuroinflammatory response induced by ethanol has been recently demonstrated [19]. Indeed, by activating the TLR4 of astroglial cells, ethanol increases the number and alters the content in inflammatory-related proteins (TLR4, nuclear factor kappa-light-chain-enhancer of activated B cells (NFκB), interleukin-1 receptor (IL-1R), caspase-1, NOD-like receptor NLRP3-inflammasome) and miRNAs (mir-146a, mir-182, mir-200b) of astrocyte-derived EVs. Furthermore, EVs from ethanol-treated astroglial cells can act by spreading neuroinflammation to neurons and compromising their survival [19]. Similarly, human studies have demonstrated that ethanol treatment is able to up-regulate several EV miRNAs in hepatocytes and liver mononuclear cells (e.g., mir-122) [20], while alcoholic hepatitis patients present a dysregulation of mir-192 and mir-30a in plasma [21].

Considering that circulating miRNAs in EVs may play an important role as biomarkers of brain diseases, the aim of this study is to evaluate whether acute alcohol intoxication differently affects the miRNA profile in the plasma EVs that derive from female and male adolescents, and if the circulating EV miRNAs could be used as biomarkers of ethanol-induced neuroinflammation in adolescence. This study reveals, for the first time, a differential gender response of plasma EVs miRNAs in humans and mice with ethanol intoxication because, while ethanol lowers the levels of anti-inflammatory miRNAs (mir-146a-5p, mir-21-5p, mir-182-5p) in human and mice female adolescents, the upregulation of these miRNAs was noted in males. These results further provide evidence that the ethanol response of the above miRNAs is similar the cerebral cortices of female and male mice, which suggests that the EVs containing miRNAs can be used as biomarkers of the neuroinflammation induced by ethanol in adolescence.

## 2. Results

### 2.1. Gender Differences in the Inflammatory-Related miRNA Profile in Plasma EVs Deriving from Ethanol-intoxicated Adolescents

The median age of females and males was 19.88 years (interquartile range (IQR) 18.0–21.0) and 20.67 years (IQR 19.0–22.0), respectively. These ages have been considered as late adolescence (ages 16–20 years) or young adulthood (ages 21–25 years) [22,23]. In addition, the biochemical analysis of plasma during the intoxication period demonstrated a median for blood alcohol levels (BALs) of 1.99 g/L (IQR 1.80–2.20) for females and 2.54 g/L (IQR 2.30–2.80) for males. No other drugs of abuse were found. These data showed a wider dispersion of BALs in females than in males. Control subjects were used, whose median ages were 22.0 years (IQR 21.2–26.2) for females and 23.0 years (IQR 21.5–25.7) for males.

By considering that miRNAs play an important role in the regulation of gene expression in many pathologies, including neuroinflammation and neurodegenerative diseases [17,24], and our studies demonstrate the action of ethanol on the miRNA profiles in murine brains [18] and in culture astroglial cells [19], we herein analyzed the levels of several miRNAs-regulated inflammatory immune responses (e.g., mir-146a-5p, mir-21-5p, mir-182-5p, mir-183-5p) in plasma EVs derived from alcohol-intoxicated female and male adolescents. 

**Figure 1:**
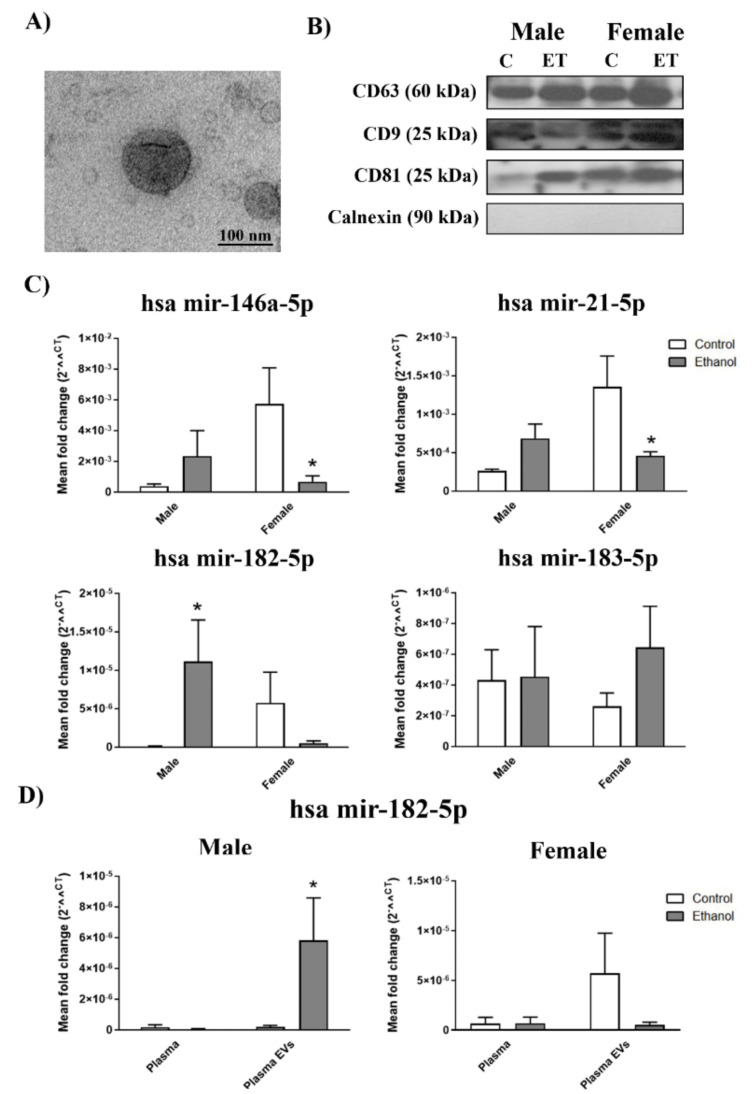
Inflammatory-related miRNA profile in the human plasma extracellular vesicles (EVs) deriving from the ethanol-intoxicated adolescents. (**A**) Human plasma EVs characterization by electron microscopy. Scale bar, 100 nm. (**B**) Representative immunoblots of the expressions of CD9, CD63, CD81, and calnexin of the human plasma EVs. (**C**) Graphs represent the expressions of the plasma EV miRNAs (mir-146a-5p, mir-21-5p, mir-182-5p, and mir-183-5p) of the human adolescent females and males after acute ethanol intoxication and the data of the corresponding healthy control individuals. (**D**) Graphs represent the mir-182-5p expression in the plasma and plasma EVs of the human adolescent females and males after acute ethanol intoxication and the data of the corresponding healthy control individuals. Data represent mean ± SEM, n = 6 independent experiments. * *p* < 0.05 compared to their respective healthy control individuals, according to the two-way ANOVA followed by the Bonferroni post hoc test.

We first characterized plasma EVs by electron microscopy and EVs markers. Thus, the employed plasma EVs had a ~100 nm-diameter, as demonstrated by electron microscopy, and were enriched in tetraspanin proteins (CD63, CD9, and CD81). They did not present cytosolic protein contamination (e.g., calnexin) (Figure 1A,B, see Figure S1 for positive control of calnexin expression). A two-way ANOVA with the Bonferroni post hoc test in Figure 1C showed that ethanol intoxication caused the levels of mir-146a-5p and mir-21-5p in the female subjects’ plasma EVs to significant lower compared to the plasma EVs samples taken from healthy female individuals. Considering mir-182-5p and mir-183-5p, we noted only a significant rise in mir-182-5p in the plasma EVs from the ethanol-intoxicated males compared to the plasma samples of the control individuals. It was interesting to note that, even though mir-183-5p lacked statistical significance in the post hoc test, an increasing tendency with ethanol treatment in females was found for it. Two-way ANOVAs were conducted to compare whether the effects of alcohol intoxication in the plasma EV miRNAs differed between female and male subjects. These analyses revealed a significant interaction between gender and ethanol intoxication in both mir-146a (F(1,20) = 5.79, *p* < 0.05), mir-21-5p (F(1,17) = 6.36, *p* < 0.05) and mir-182-5p (F(1,24) = 6.28, *p* < 0.05), but not in mir-183-5p (F(1,20) = 0.58, *p* > 0.05) (Figure 1C).

Considering that EV miRNAs are more stable in peripheral circulation than plasma miRNAs, we compared the miRNA expression between plasma and the plasma EVs. Figure 1D shows how mir-182-5p expression was very low (almost undetectable) in the plasma of the healthy and ethanol intoxicated individuals; in contrast we noted a significant increase in the plasma EVs of the ethanol intoxicated male subjects and a non-significant decrease in the ethanol intoxicated female individuals. No significant differences were obtained between plasma and the plasma EVs in mir-146a-5p, mir-21-5p, and mir-183-5p (Figure S2). Two-way ANOVAs were used to demonstrate differences in the miRNA expression between plasma and plasma EVs. A significant interaction between sample (plasma or plasma EVs) and treatment in the male subjects (F(1,12) = 8.12, *p* < 0.05), but not in the female individuals (F(1,20) = 1.61, *p* > 0.05), was noted.

### 2.2. Gender Differences in the miRNA Inflammatory-Related Profile in the Plasma EVs Deriving from the Ethanol-Treated Adolescent Mice

**Figure 2:**
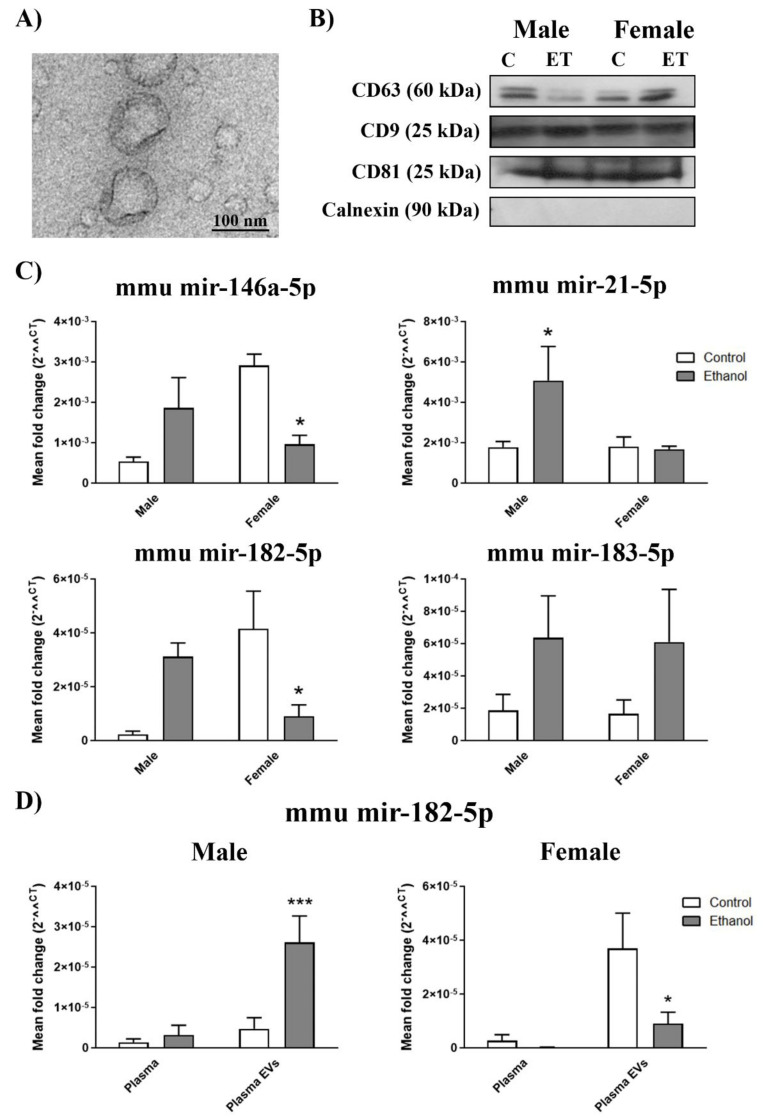
Inflammatory-related miRNA profile in the plasma EVs deriving from the ethanol-treated adolescent wild type (WT) mice. (**A**) Murine plasma EVs characterization by electron microscopy. Scale bar, 100 nm. (**B**) Representative immunoblots of the expressions of CD9, CD63, CD81, and calnexin of the murine plasma EVs. (**C**) Graphs represent the expressions of plasma EV miRNAs (mir-146a-5p, mir-21-5p, mir-182-5p, and mir-183-5p) after ethanol or saline treatment in the adolescent female and male WT mice (postnatal day (PND) 44). (**D**) Graphs represent the mir-182-5p expression in plasma and plasma EVs after the ethanol or saline treatment in the adolescent female and male WT mice (PND 44). Data represent mean ± SEM, *n* = 6 independent experiments. * *p* < 0.05, *** *p* < 0.001 compared to their respective control counterparts, according to the two-way ANOVA followed by the Bonferroni post hoc test.

To further investigate the gender differences in the circulating EV miRNAs and the involvement of the TLR4 immune response in these effects, we used an experimental model of intermittent ethanol treatment in the wild type (WT) and TLR4-knockout (KO) adolescent mice to mimic the adolescent individuals’ drinking pattern. Both female and male mice showed similar BALs and ethanol clearance post-injection, as previously described [17]. According to human plasma EVs, Figure 2A,B shows the plasma EVs characterization in mice (see Figure S1 for positive control of calnexin expression). The two-way ANOVA with the Bonferroni post hoc test shown in Figure 2C revealed that intermittent ethanol treatment significantly lowered the mir-146a-5p and mir-182-5p levels in the female subjects’ plasma EVs and significantly increased the mir-21-5p levels in the male mice. Although the plasma EVs levels in mir-182-5p of the ethanol intoxicated males and in mir-183-5p of the ethanol, intoxicated female and male subjects were not significant compared to the healthy individuals, there was a tendency to differ when compared to their control counterparts (Figure 2C). Two-way ANOVAs were conducted to determine gender differences in the plasma EV miRNAs from the ethanol-treated WT mice. These analyses revealed a significant interaction between gender and ethanol treatment in mir-146a-5p (F(1,23) = 14.46, *p* < 0.05), mir-21-5p (F(1,27) = 3.38, *p* < 0.05) and mir-182-5p (F(1,26) = 13.73, *p* < 0.05), but no significant interaction appeared in mir-183-5p (F(1,26) = 0.0001, *p* > 0.05).

Similarly to human data, we also compared the miRNAs expression between the plasma and plasma EVs in mice (Figure 2D). Once again, mir-182-5p expression was almost undetectable in the plasma of the saline- and ethanol-treated mice; in contrast in the plasma EVs levels of mir-182-5p, we noted a significant increase in the ethanol-treated males and a significant decrease in the ethanol-treated females versus their control counterparts (Figure 2D). No significant differences between plasma and plasma EVs were obtained in mir-146a-5p, mir-21-5p, and mir-183-5p (Figure S3). Two-way ANOVAs were used to demonstrate differences in the miRNA expression between plasma and plasma EVs. A significant interaction between sample (plasma or plasma EVs) and treatment in the male subjects (F(1,28) = 9.13, *p* < 0.05) appeared, while this interaction was not significant in females (F(1,31) = 3.11, *p* > 0.05), but was significant for sample (F(1,31) = 8.95, *p* < 0.05) and treatment (F(1,31) = 4.47, *p* < 0.05).

**Figure 3:**
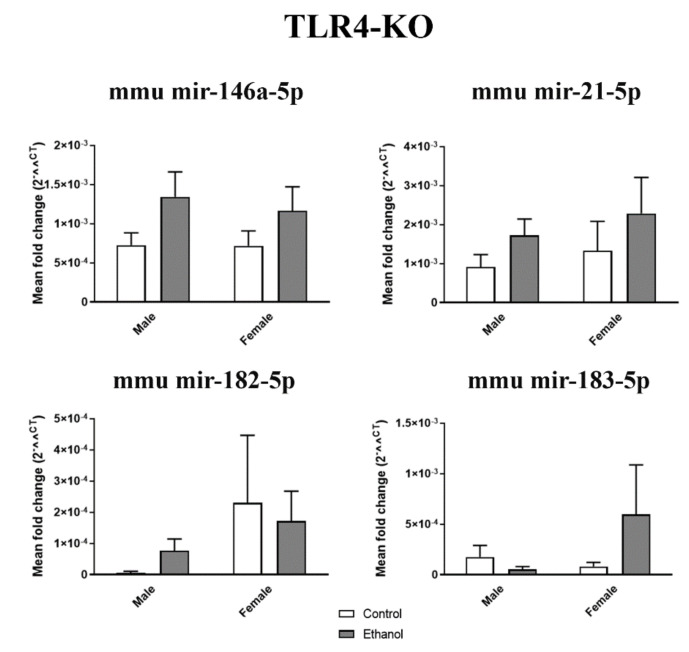
Inflammatory-related miRNA profile in the plasma EVs deriving from the ethanol-treated adolescent toll-like receptor 4 (TLR4)-knockout (KO) mice. Graphs represent the expression of plasma EV miRNAs (mir-146a-5p, mir-21-5p, mir-182-5p, and mir-183-5p) after the ethanol or saline treatment in the adolescent female and male TLR4-KO mice (PND 44). Data represent mean ± SEM, *n* = 6 independent experiments. No significant differences were shown, according to the two-way ANOVA followed by the Bonferroni post hoc test.

We also assessed the levels of plasma EV miRNAs from the saline- and ethanol-treated TLR4-KO female and male mice. As shown in Figure 3, no significant changes were noted in mir-146a-5p (F(1,20) = 0.11, *p* > 0.05), mir-21-5p (F(1,20) = 0.01, *p* > 0.05), mir-182-5p (F(1,19) = 0.36, *p* > 0.05), and mir-183-5p (F(1,18) = 1.95, *p* > 0.05).

### 2.3. Ethanol Binge Drinking Induces Changes in Inflammatory-miRNAs and Their Target Genes in Mice Cerebral Cortices

**Figure 4:**
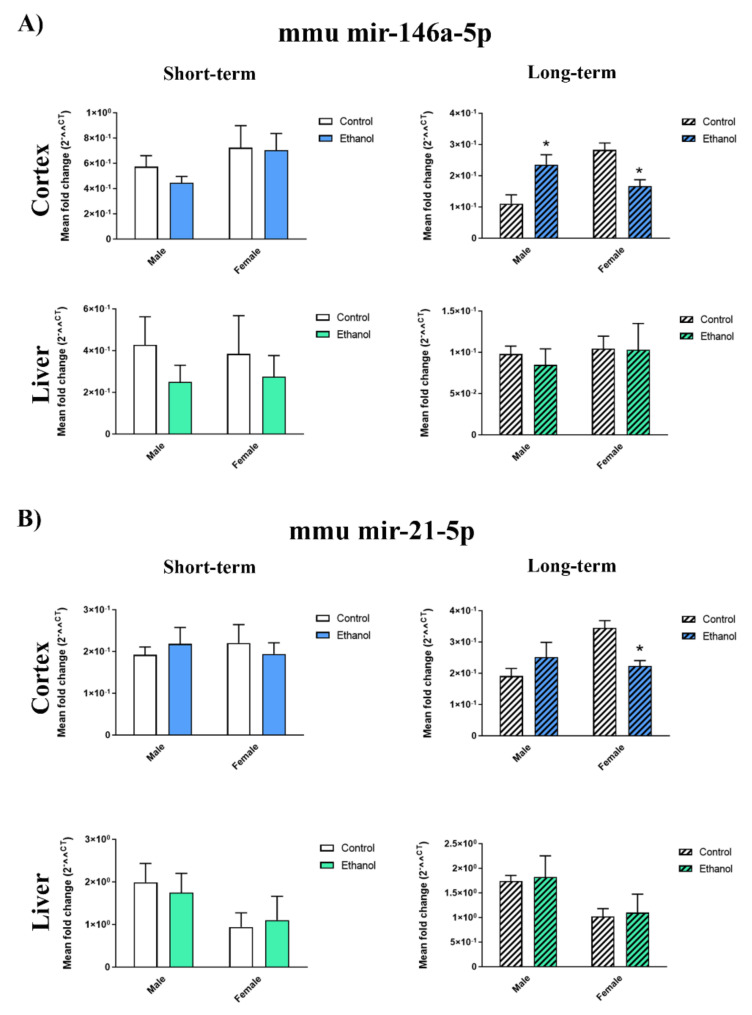
Effects of ethanol in the inflammatory-related miRNA profile in the cerebral cortices and livers of adolescent WT mice. Graphs represent the expression of (**A**) mir-146a-5p and (**B**) mir-21-5p after a 24-h and 2-week withdrawal period of ethanol or saline treatment in the female and male adolescent WT mice (PND 44 and PND 58). Data represent mean ± SEM, *n* = 6 independent experiments. * *p* < 0.05 compared to their respective control counterparts, according to the two-way ANOVA followed by the Bonferroni post hoc test.

We next evaluated whether the gender differences found in the inflammatory miRNAs in the plasma EVs shown in the ethanol-treated WT adolescent mice were associated with the different expressions of these molecules in cerebral cortex and liver, and whether these changes maintained in adulthood. To answer this question, we measured the levels mir-146a-5p and mir-21-5p in the whole cerebral cortices and livers of WT mice after a 24-h and 2-week withdrawal period (short- and long-term ethanol effects). Figure 4 shows that whereas the intermittent ethanol treatment increased the mir-146a-5p levels in the brain cortices of the young adult male mice exposed to ethanol in adolescence (long-term ethanol effects), the mir-146a-5p and mir-21-5p levels lowered in the brain cortices of the young adult female mice. In addition, no significant differences were noted between the saline- and ethanol-treated mice in the brain cortex for the short-term ethanol effects and in the liver for the short- and long-term effects. Two-way ANOVAs revealed a significant interaction between gender and ethanol treatment in mir-146a-5p (F(1,12) = 21.60, *p* < 0.05) and mir-21-5p (F(1,16) = 9.28, *p* < 0.05) in the brain cortices of the young adult mice exposed to ethanol in adolescence.

**Figure 5:**
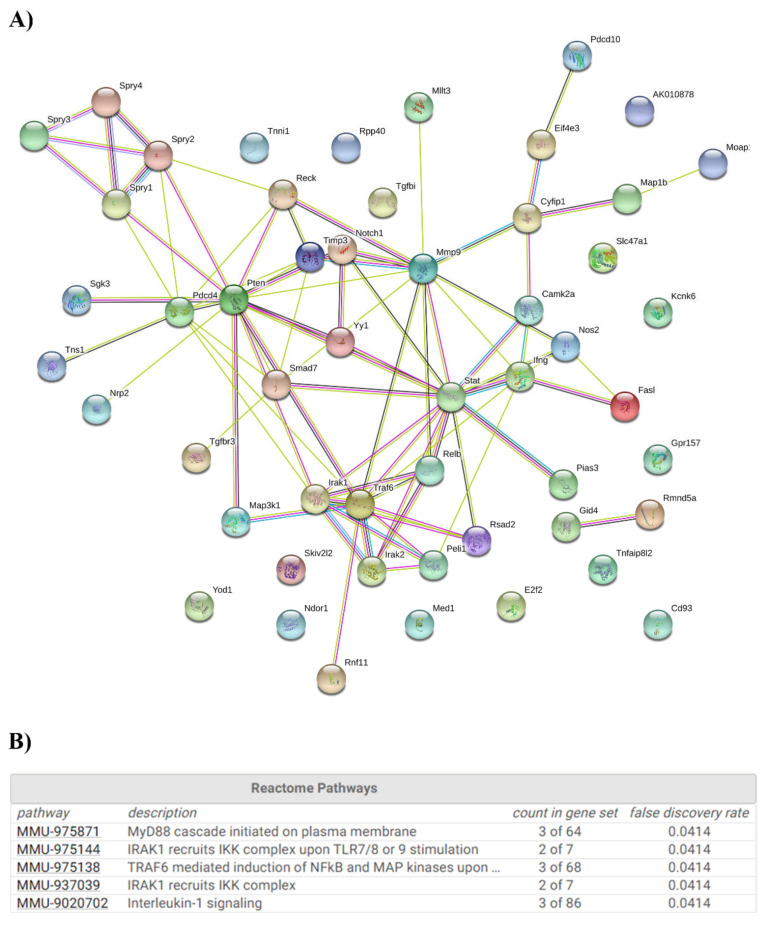
Functional analysis of mir-146a-5p and mir-21-5p. (**A**) Protein–protein interaction for the predicted genes of each miRNA. (**B**) The principal Reactome pathways were modulated by the STRING protein–protein network interaction analysis, performed with the STRING webserver, and *p*-values are represented as (−log(*p*-value)).

Given the significant interaction between ethanol treatment and gender in the specific miRNA expression, we decided to perform a functional analysis to determine the possible targets of these miRNAs and their role in the brain. For this aim, we performed a bioinformatic analysis based on two steps. First, we determined the targets potentially regulated by mir-146a-5p and mir-21a-5p using the miRNet platform (www.mirnet.ca) (Table S3). Then, with the target genes obtained, we assessed the metabolic pathways that could be affected by the dysregulation of these genes. For this objective, a functional enrichment was carried out using with the STRING platform (www.string.es) (Figure 5A). The results obtained in Figure 5B illustrated a significant effect in the Reactome pathways related to inflammation, including such as the MyD88 signaling cascade; the NFκβ route via IRAK1 and TRAF6, among others.

**Figure 6:**
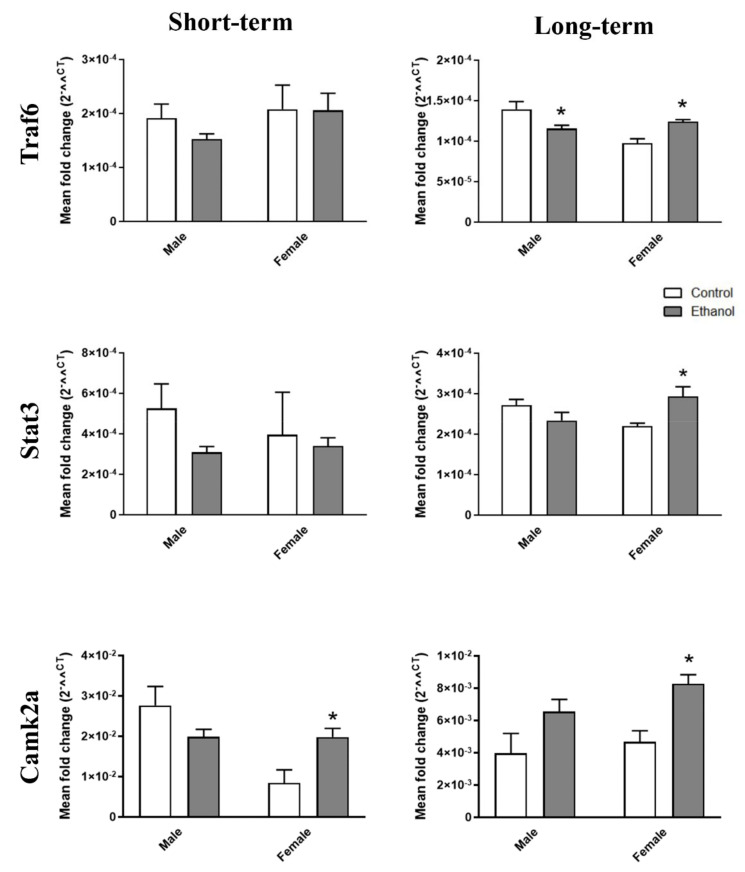
Effects of ethanol on the target gene profile of mir-146a-5p and mir-21-5p in the cerebral cortices of adolescent WT mice. Graphs represent the expression of Traf6, Stat3, and Camk2a after a 24-h and 2-week withdrawal period of ethanol or saline treatment in the female and male adolescent WT mice (PND 44 and PND 58). Data represent mean ± SEM, *n* = 6 independent experiments. * *p* < 0.05 compared to their respective control counterparts, according to the two-way ANOVA followed by the Bonferroni post hoc test.

Then we also analyzed inflammatory- and TLR4-related genes, such as Traf6, Stat3, and Camk2a, which are modulated by mir-146a-5p and mir-21-5p [25,26]. Figure 6 depicts that whereas the intermittent ethanol treatment lowered the Traf6 levels in brain cortices of the young adult male mice exposed to ethanol in adolescence (long-term ethanol effects), the Traf6, Stat3, and Camk2a levels rose in the brain cortices of the young adult female mice. Furthermore, the Traf6 levels increased in the livers of the male adolescent mice exposed to ethanol (Figure S5). The Camk2a levels increased in the ethanol-treated female mice compared to the control mice for the short- and long-term effects (Figure 6). No significant differences were noted in most of the comparisons made of the saline- and ethanol-treated mice in the brain cortex for the short-term and in the liver for the short-and long-term effects (Figure 6 and Figure S5). Two-way ANOVAs revealed a significant interaction between gender and ethanol treatment in Traf6 (F(1,16) = 13.83, *p* < 0.05), Stat3 (F(1,15) = 10.03, *p* < 0.05), and Camk2a (F(1,20) = 9.12, *p* < 0.05) in the brain cortices of the young adult mice exposed to ethanol in adolescence.

## 3. Discussion

Our recent findings demonstrated that human female adolescents are more vulnerable than males to the inflammatory effects of binge ethanol drinking because, at an equivalent BAL, the female adolescents displayed an activation of the TLR4 immune response with higher levels of plasma cytokines and chemokines than the male adolescents after acute alcohol intoxication [17]. As we have previously shown that glial EVs participate in extending TLR4 neuroinflammation and neuronal dysfunctions induced by ethanol [19], and that EVs are very stable in peripheral circulation [6], we analyzed whether acute alcohol intoxication differently affected the miRNA profile in the plasma EVs deriving from female and male adolescents, and if circulating EV miRNAs could be used as biomarkers of neuroinflammation induced by ethanol in adolescence. We herein demonstrate, for the first time, that whereas both human and mice female adolescents had lower levels of anti-inflammatory miRNAs (mir-146a-5p, mir-21-5p, and mir-182-5p) in plasma EVs after ethanol intoxication, males had higher miRNAs levels. We also show that the brain cortices from the female young adult mice had lower levels of these miRNAs, and a higher expression of their inflammatory target genes (Traf6, Stat3, and Camk2a) than the young adult males exposed to ethanol in adolescence. This scenario suggests that EV miRNAs could be used as biomarkers of neuroinflammation induced by ethanol in adolescence.

In the last decade, plasma or serum miRNAs have emerged as diagnostic biomarkers for disease, but their use is still limited given the low concentrations in these fluids [27]. However, EVs show miRNAs enrichment, and are more stable in peripheral circulation than the plasma miRNAs because EVs protect them from degradation [6]. Accordingly, our results revealed that one miRNA (e.g., mir-182-5p), with a low expression in plasma EVs, was almost undetectable in the plasma samples. As for the function of EV miRNAs, recent findings have demonstrated the role of EV miRNAs in neurodegenerative diseases [8] and inflammatory processes [9]. These results revealed that human female adolescents had lower levels of the anti-inflammatory miRNAs, mir-146a-5p, mir-21-5p, and mir-182-5p in plasma EVs after ethanol intoxication, whereas the levels of these miRNAs were higher in the male adolescents. Several reports correlate the upregulation of these miRNAs with a depression of the innate immune response. For instance, mir-146a negatively modulates T-cell adhesion and blocks M1 macrophage activation by inhibiting the NFκB pathway, which could be used as a therapeutic strategy in some neurodegenerative diseases (e.g., Alzheimer’s disease) [28,29,30]. A recent report has also demonstrated that the co-incubation of mir-182 in LPS-treated cells diminishes the release of pro-inflammatory cytokines, such as IL-6, IL-1β and TNF-α [31]. Moreover, other studies have demonstrated that mir-21 participates in inflammatory processes that promote the macrophage engulfment of apoptotic cells [8], while others have demonstrated the involvement of mir-21 in inflammation and apoptosis by targeting genes, such as PTEN, BCL2, Il-12, and STAT3, among others [25,32]. Although it is uncertain whether there is difference in EV secretion mechanisms between female and male after ethanol intoxication, the lower anti-inflammatory miRNAs levels in the plasma-derived EVs after ethanol intoxication in the female versus male adolescents could be associated with a better inflammatory immune response in the female adolescents than in their male counterparts. This finding suggests that adolescent females could be more vulnerable than males to the inflammatory effects of binge ethanol drinking. Similarly, human studies have revealed that female adolescents present higher levels of plasma inflammatory molecules than male adolescents [17,33], which correlates with worse memory and executive functioning task score for female binge drinkers, but no for male binge drinkers [33].

According to human data, our results also demonstrated that the miRNAs in plasma EVs were downregulated in the ethanol-treated adolescent female mice and were upregulated in the male mice. These effects were associated with the TLR4 response because the changes in the miRNA profile in the plasma EVs of both the female and male adolescent mice were abolished in the TLR4-deficient mice, which suggests the involvement of the TLR4 immune response in these events. We have previously demonstrated that alcohol is capable of activating the TLR4 signaling response in glial cells [13,14] by triggering neuroinflammation and neural damage in adolescent animals exposed to alcohol binge drinking [15]. Furthermore, the role of TLR4 has been recently involved in mir-183 cluster dysregulation (mir182-mir183-mir96), the mir-200a/b expression induced by chronic ethanol treatment in mice cerebral cortices [18], and the increased release of astrocyte-derived EVs and their content enriched in inflammation-related proteins and miRNAs ethanol-treated astrocyte-derived EVs [19].

As EVs may play an important role as carriers of miRNAs through the blood–brain barrier into the circulatory system [6], the circulating miRNAs in EVs can be considered good biomarkers of brain diseases. For instance, multiple sclerosis patients reveal a dysregulation of several exosomal miRNAs in plasma compared to healthy age- and gender-matched controls [34], and the plasma exosomes isolated from Alzheimer’s disease patients reveal a combination of miRNAs that could be useful in early diagnostics [35]. The present study also shows how similar changes in the miRNAs from the plasma EVs of the ethanol-treated adolescent mice were obtained in cerebral cortices (e.g., mir-146a-5p and mir-21-5p) of young adult mice exposed to ethanol in adolescence, which suggests that these plasma EV miRNAs could be useful biomarkers of ethanol-induced neuroinflammation in adolescence. By a bioinformatics analysis, we noted that mir-146a-5p and mir-21-5p performed a joint action with some of the targets involved in signaling molecules associated with the TLR4 and IL-1 signaling pathways. Moreover, these changes are associated with a higher expression of inflammatory- and TLR4-related genes (e.g., Traf6, Stat3, and Camk2a) [26,36] in the cerebral cortices of ethanol-treated mice. The expression of these genes Traf6 and Stat3 activates signaling pathways, which leads to the release of cytokines and chemokines [37,38]. Camk2a has been shown to be a calcium-dependent protein kinase involved in inflammation through the activation of p-TAK, p-JNK and NLRP3 inflammasome [39]. The expression of these miRNAs and their target genes in the brain, cortices of ethanol-treated adolescent mice could trigger an inflammatory immune response in microglia and astroglia [13,14], which leads to the production of both pro-inflammatory cytokines/chemokines and EVs-containing inflammatory molecules by spreading neuroinflammation, and causing brain damage and long-term cognitive and behavioral dysfunctions [15,16]. Indeed a recent report shows that serum exosomes of LPS-treated mice administered to naïve animals induced not only microglial and astroglial activation, but also levels of both proinflammatory cytokines and mir-155 in the brain [40]. Conversely, although our findings show that ethanol is unable to induce short-term alterations in both miRNAs and their target genes in the livers or brain cortices of adolescent animals, we considered that other inflammatory-related miRNAs and target gene pathways could be involved in ethanol effects, as demonstrated in the brain after chronic ethanol treatment [18], or in the plasma of alcoholic hepatitis patients [21].

These results indicate, for the first time, that female and male adolescents differ in terms of the effects of ethanol associated with the expression of inflammatory-related miRNAs in plasma EVs, which supports the notion of females vs. males being more to the inflammatory effects of binge alcohol drinking. These findings also support that circulating EV miRNAs are good candidates as biomarkers to detect neuroinflammation and brain damage induced by ethanol in adolescence.

## 4. Materials and Methods

### 4.1. Human Subjects

Our clinical sample included 18 adolescent and young adults (50% females) who were admitted to the Emergency Department of the University Hospital of Salamanca (Salamanca, Spain) with moderate to severe acute alcohol intoxication, as previously reported [17]. The individuals’ clinical, epidemiological, and analytical characteristics are shown in Table 1. In addition, eighteen healthy controls (nine males, nine females) were included in the study, and were recruited among medical and nursing students. The controls did not consume alcohol apart from light sporadic drinking, and reported neither drinking alcohol during the 72-h period prior to drawing blood nor any binge drinking episodes in the last 3 months. These subjects showed normal hematological and plasma biochemical parameters and reported no chronic or acute illness. The study was conducted in accordance with the Declaration of Helsinki and was approved by the Ethics Committee of the University Hospital of Salamanca (22 November 2012). All the subjects gave their informed consent for inclusion before participating in the study. Blood samples were obtained from the patients upon admission for use in standard care, and also for research purposes. These blood samples were used to determine the BAL, blood count and liver function tests, and were then snap-frozen in liquid nitrogen and stored at −80 °C until used. Samples were processed and analyzed for this study only after patients were able to provide informed consent.

### 4.2. Animals and Treatment

C57/BL6 wild type (WT) and TLR4-knockout (TLR4-KO) (C57/BL6 background, kindly provided by Dr. S. Akira, Osaka, Japan) mice were used. Animals were distributed into 3–4 animals per cage, separated by genotype. They were maintained with water and solid diet ad libitum under controlled conditions of temperature (23 °C), humidity (60%), and light/dark cycles (12 h/12 h). All of the experimental procedures were carried out in accordance with the guidelines approved by European Communities Council Directive (86/609/ECC) and Spanish Royal Decree 1201/2005 with the approval of the Ethical Committee of Animal Experimentation of the Príncipe Felipe Research Centre (Valencia, Spain) on 19 June, 2019 (Project identification code: 2019-08).

For the binge ethanol treatment, morning doses (9–10 a.m.) of either saline or 25% (*v*/*v*) ethanol (3 g/kg) in isotonic saline were administered intraperitoneally to 30-day-old mice on 2 consecutive days with 2-day gaps without injections for 2 weeks (PND 30 to PND 43), as previously described [41]. Animals were anesthetized 24 h after the last (8th) ethanol or saline administration (PND 44, short-term ethanol effects) or after 2 weeks upon ethanol or saline administration (PND 58, long-term ethanol effects). Whole blood was collected from the hepatic portal vein. After centrifugation, the separated plasma, cerebral cortices, and livers dissected were snap-frozen in liquid nitrogen and stored at −80 °C until used.

### 4.3. EVs Isolation from Human and Mouse Plasma

The isolation of the EVs derived from plasma was carried out following the manufacturer’s instructions (Total Exosome Isolation Kit, Invitrogen, Vilnius, Lithuania). Then, 200 μL of initial plasma were used for the EVs isolation, which were collected and frozen to −80 °C until processed. 

### 4.4. EVs Characterization by Transmission Electron Microscopy

Freshly isolated EVs were fixed with 2% paraformaldehyde and prepared as previously described [42]. Preparations were examined under a transmission FEI Tecnai G2 Spirit electron microscope (FEI Europe, Eindhoven, The Netherlands) using a Morada digital camera (Olympus Soft Image Solutions GmbH, Münster, Germany).

### 4.5. Western Blot Analysis of EVs

For the Western blot analysis, equal µgs of EVs were separated in SDS-PAGE gels and transferred to PVDF membranes, as previously described [14]. The used antibodies were: anti-CD63, anti-CD9, anti-CD81, and anti-calnexin (Santa Cruz Biotechnology, Madrid, Spain). Membranes were incubated with the respective anti-HRP secondary antibodies and developed by the ECL system (ECL Plus; Thermo Fisher Scientific, Illinois, USA). Band intensity was quantified using the ImageJ 1.44p analysis software. The densitometric analysis is shown in arbitrary units. Figure S2 includes the whole membrane of each protein expression.

### 4.6. RNA Isolation, Reverse Transcription, and Quantitative RT-PCR

The total RNA of plasma EVs and plasma was isolated from initial 200 μl of plasma, following the manufacturer’s instructions (Total Exosome RNA Isolation Kit, Invitrogen, Vilnius, Lithuania). Likewise, whole cerebral cortices and livers were lysed in 1 mL of Tri-Reagent solution (Sigma-Aldrich, Madrid, Spain) and RNA was isolated according to the manufacturer’s instructions. Total mRNA and total miRNA were reverse-transcribed by the NZY First-Strand cDNA Synthesis Kit (NZYTech, Lda. Genes and Enzymes, Lisboa, Portugal) and TaqMan Advanced miRNA Assays (Thermo Fisher Scientific, Illinoi, USA), respectively.

Quantitative two-step RT-PCR (real-time reverse transcription) was performed with the Light-Cycler 480 detection System (Roche Diagnostics, Madrid, Spain). Genes were amplified using the AceQ Universal SYBR qPCR Master Mix following the manufacturer’s instructions (Vazyme Biotech Co., Ltd., Nanjing, China). The mRNA level of housekeeping gene cyclophilin A was used as an internal control for the normalization of analyzed genes. Specific miRNAs assays were amplified by the TaqMan Fast Advanced Master Mix (Thermo Fisher Scientific, Illinois, USA) and mir-451 was used as an internal control for both the plasma EVs and tissue samples (its stability is shown in Figure S4). Data were analyzed by the LightCycler 480 relative quantification software. The nucleotide sequences of the used primers and miRNAs assays are detailed in the Supplementary Materials (Tables S1 and S2).

## Figures and Tables

**Table 1:** Characteristics of individuals with acute alcohol intoxication.

	Males (*n* = 9)	Females (*n* = 9)
Age (years)	20.67 (1.36)	19.88 (1.50)
BALs (g/L)	2.54 (0.14)	1.99 (0.09)
Aspartate aminotransferase levels (IU/L)	29.62 (6.80)	18.88 (1.30)
Alanine aminotransferase levels (IU/L)	24.12 (9.46)	15.22 (1.43)
Alkaline phosphatase levels (IU/L)	78.62 (8.81)	61.00 (3.18)
*γ*- glutamyl transpeptidase levels (IU/L)	28.88 (10.67)	12.66 (1.59)
White blood cell count/μL	8936.67 (822.90)	8935.56 (810.54)
Individuals who reported weekend drinking (%) *	5 (71.4%)	8 (88.9%)

Quantitative variables are presented as the mean (SEM) and qualitative variables are presented as absolute frequencies (percentage). IU, international units. BALs: blood alcohol levels. * Two male individuals refused to answer the questionnaire regarding drinking patterns.

## Supplementary Materials

Supplementary Materials can be found at https://www.mdpi.com/1422-0067/21/18/6730/s1.

## References

[1] Patel N., Hoang D., Miller N., Ansaloni S., Huang Q., Rogers J.T., Lee J.C., Saunders A.J. (2008). MicroRNAs can regulate human APP levels. Mol. Neurodegener..

[2] Wang W.X., Rajeev B.W., Stromberg A.J., Ren N., Tang G., Huang Q., Rigoutsos I., Nelson P.T. (2008). The expression of microRNA miR-107 decreases early in Alzheimer’s disease and may accelerate disease progression through regulation of β-site amyloid precursor protein-cleaving enzyme 1. J. Neurosci..

[3] Balusu S., Van Wonterghem E., De Rycke R., Raemdonck K., Stremersch S., Gevaert K., Brkic M., Demeestere D., Vanhooren V., Hendrix A. (2016). Identification of a novel mechanism of blood-brain communication during peripheral inflammation via choroid plexus-derived extracellular vesicles. EMBO Mol. Med..

[4] Wang J., Sun X., Zhao J., Yang Y., Cai X., Xu J., Cao P. (2017). Exosomes: A novel strategy for treatment and prevention of diseases. Front. Pharmacol..

[5] Mathivanan S., Ji H., Simpson R.J. (2010). Exosomes: Extracellular organelles important in intercellular communication. J. Proteom..

[6] Chen Y., Xie Y., Xu L., Zhan S., Xiao Y., Gao Y., Wu B., Ge W. (2017). Protein content and functional characteristics of serum-purified exosomes from patients with colorectal cancer revealed by quantitative proteomics. Int. J. Cancer.

[7] Cheng L., Quek C.Y.J., Sun X., Bellingham S.A., Hill A.F. (2013). The detection of microRNA associated with alzheimer’s disease in biological fluids using next-generation sequencing technologies. Front. Genet..

[8] Slota J.A., Booth S.A. (2019). MicroRNAs in neuroinflammation: Implications in disease pathogenesis, biomarker discovery and therapeutic applications. Non-Coding RNA.

[9] Gupta A., Pulliam L. (2014). Exosomes as mediators of neuroinflammation. J. Neuroinflamm..

[10] Alfonso-Loeches S., Guerri C. (2011). Molecular and behavioral aspects of the actions of alcohol on the adult and developing brain. Crit. Rev. Clin. Lab. Sci..

[11] Toga A.W., Thompson P.M., Sowell E.R. (2006). Mapping brain maturation. Trends Neurosci..

[12] Jacobus J., Tapert S.F. (2013). Neurotoxic Effects of Alcohol in Adolescence. Annu. Rev. Clin. Psychol..

[13] Alfonso-Loeches S., Pascual-Lucas M., Blanco A.M., Sanchez-Vera I., Guerri C. (2010). Pivotal Role of TLR4 Receptors in Alcohol-Induced Neuroinflammation and Brain Damage. J. Neurosci..

[14] Fernandez-Lizarbe S., Pascual M., Guerri C. (2009). Critical Role of TLR4 Response in the Activation of Microglia Induced by Ethanol. J. Immunol..

[15] Montesinos J., Pascual M., Pla A., Maldonado C., Rodríguez-Arias M., Miñarro J., Guerri C. (2015). TLR4 elimination prevents synaptic and myelin alterations and long-term cognitive dysfunctions in adolescent mice with intermittent ethanol treatment. Brain. Behav. Immun..

[16] Montesinos J., Alfonso-Loeches S., Guerri C. (2016). Impact of the Innate Immune Response in the Actions of Ethanol on the Central Nervous System. Alcohol. Clin. Exp. Res..

[17] Pascual M., Montesinos J., Marcos M., Torres J.L., Costa-Alba P., García-García F., Laso F.J., Guerri C. (2017). Gender differences in the inflammatory cytokine and chemokine profiles induced by binge ethanol drinking in adolescence. Addict. Biol..

[18] Ureña-Peralta J.R., Alfonso-Loeches S., Cuesta-Diaz C.M., García-García F., Guerri C. (2018). Deep sequencing and miRNA profiles in alcohol-induced neuroinflammation and the TLR4 response in mice cerebral cortex. Sci. Rep..

[19] Ibáñez F., Montesinos J., Ureña-Peralta J.R., Guerri C., Pascual M. (2019). TLR4 participates in the transmission of ethanol-induced neuroinflammation via astrocyte-derived extracellular vesicles. J. Neuroinflamm..

[20] Momen-Heravi F., Bala S., Kodys K., Szabo G. (2015). Exosomes derived from alcohol-treated hepatocytes horizontally transfer liver specific miRNA-122 and sensitize monocytes to LPS. Sci. Rep..

[21] Momen-Heravi F., Saha B., Kodys K., Catalano D., Satishchandran A., Szabo G. (2015). Increased number of circulating exosomes and their microRNA cargos are potential novel biomarkers in alcoholic hepatitis. J. Transl. Med..

[22] Brown S.A., McGue M., Maggs J., Schulenberg J., Hingson R., Swartzwelder S., Martin C., Chung T., Tapert S.F., Sher K. (2008). A developmental perspective on alcohol and youths 16 to 20 years of age. Pediatrics.

[23] Masten A.S., Faden V.B., Zucker R.A., Spear L.P. (2009). A developmental perspective on underage alcohol use. Alcohol Res. Health.

[24] Pascual M., Ibáñez F., Guerri C. (2020). Exosomes as mediators of neuron-glia communication in neuroinflammation. Neural Regen. Res..

[25] Sen C.K., Roy S. (2012). MicroRNA 21 in tissue injury and inflammation. Cardiovasc. Res..

[26] Alexander M., Hu R., Runtsch M.C., Kagele D.A., Mosbruger T.L., Tolmachova T., Seabra M.C., Round J.L., Ward D.M., O’Connell R.M. (2015). Exosome-delivered microRNAs modulate the inflammatory response to endotoxin. Nat. Commun..

[27] Huang M., Gonzalez R.R., Lillard J., Bond V.C. (2017). Secretion modification region-derived peptide blocks exosome release and mediates cell cycle arrest in breast cancer cells. Oncotarget.

[28] Cui J.G., Li Y.Y., Zhao Y., Bhattacharjee S., Lukiw W.J. (2010). Differential regulation of Interleukin-1 Receptor-associated Kinase-1 (IRAK-1) and IRAK-2 by microRNA-146a and NF-κB in stressed human astroglial cells and in Alzheimer disease. J. Biol. Chem..

[29] He X., Tang R., Sun Y., Wang Y.G., Zhen K.Y., Zhang D.M., Pan W.Q. (2016). MicroR-146 blocks the activation of M1 macrophage by targeting signal transducer and activator of transcription 1 in hepatic schistosomiasis. EBioMedicine.

[30] Wu D., Cerutti C., Lopez-Ramirez M.A., Pryce G., King-Robson J., Simpson J.E., van der Pol S.M., Hirst M.C., de Vries H.E., Sharrack B. (2015). Brain endothelial miR-146a negatively modulates T-cell adhesion through repressing multiple targets to inhibit NF-κB activation. J. Cereb. Blood Flow Metab..

[31] Zhu M., Li Y., Sun K. (2018). MicroRNA-182-5p inhibits inflammation in LPS-treated RAW264.7 cells by mediating the TLR4/NF-κB signaling pathway. Int. J. Clin. Exp. Pathol..

[32] Buscaglia L.E.B., Li Y. (2011). Apoptosis and the target genes of microRNA-21. Chin. J. Cancer.

[33] Orio L., Antón M., Rodríguez-Rojo I.C., Correas Á., García-Bueno B., Corral M., de Fonseca F.R., García-Moreno L.M., Maestú F., Cadaveira F. (2018). Young alcohol binge drinkers have elevated blood endotoxin, peripheral inflammation and low cortisol levels: Neuropsychological correlations in women. Addict. Biol..

[34] Siegel S.R., MacKenzie J., Chaplin G., Jablonski N.G., Griffiths L. (2012). Circulating microRNAs involved in multiple sclerosis. Mol. Biol. Rep..

[35] Yang T.T., Liu C.G., Gao S.C., Zhang Y., Wang P.C. (2018). The Serum Exosome Derived MicroRNA−135a, −193b, and −384 Were Potential Alzheimer’s Disease Biomarkers. Biomed. Environ. Sci..

[36] Sadler A.J., Suliman B.A., Yu L., Yuan X., Wang D., Irving A.T., Sarvestani S.T., Banerjee A., Mansell A.S., Liu J.P. (2015). The acetyltransferase HAT1 moderates the NF-κB response by regulating the transcription factor PLZF. Nat. Commun..

[37] Sato S., Sugiyama M., Yamamoto M., Watanabe Y., Kawai T., Takeda K., Akira S. (2003). Toll/IL-1 receptor domain-containing adaptor inducing IFN-beta (TRIF) associates with TNF receptor-associated factor 6 and TANK-binding kinase 1, and activates two distinct transcription factors, NF-kappa B and IFN-regulatory factor-3, in the Toll-like receptor signaling. J. Immunol..

[38] Yu H., Pardoll D., Jove R. (2009). STATs in cancer inflammation and immunity: A leading role for STAT3. Nat. Rev. Cancer.

[39] Zhou K., Enkhjargal B., Xie Z., Sun C., Wu L., Malaguit J., Chen S., Tang J., Zhang J., Zhang J.H. (2018). Dihydrolipoic acid inhibits lysosomal rupture and NLRP3 through lysosome-associated membrane Protein-1/Calcium/Calmodulin-Dependent Protein Kinase II/TAK1 pathways after subarachnoid hemorrhage in rat. Stroke.

[40] Li J.J., Wang B., Kodali M.C., Chen C., Kim E., Patters B.J., Lan L., Kumar S., Wang X., Yue J. (2018). In vivo evidence for the contribution of peripheral circulating inflammatory exosomes to neuroinflammation. J. Neuroinflammation.

[41] Pascual M., Guerri C. (2007). The peptide NAP promotes neuronal growth and differentiation through extracellular signal-regulated protein kinase and Akt pathways, and protects neurons co-cultured with astrocytes damaged by ethanol. J. Neurochem..

[42] Théry C., Amigorena S., Raposo G., Clayton A. (2006). Isolation and Characterization of Exosomes from Cell Culture Supernatants and Biological Fluids. Curr. Protoc. Cell Biol..

