# Supplementary material for: Circulating MicroRNAs in Extracellular Vesicles as Potential Biomarkers of Alcohol-Induced Neuroinflammation in Adolescence: Gender Differences
Source: Int J Mol Sci. 2020 Sep 14;21(18):6730. doi: 10.3390/ijms21186730 (PMC7555060; doi:10.3390/ijms21186730)
Supplement: Supplementary file 1 [file ijms-21-06730-s001.pdf]

International Journal of
Molecular Sciences

MDPI

Supplementary Material

Table S1. Nucleotide sequences of the primers used for RT-PCR of micro-RNAs.

MicroRNA	Chromosome Location	Accession Number (#)	Mature Primer sequences (5′ to 3′)
**mmu-mir-146a-5p**	Chr.5: 160485352 - 160485450 [+] on Build GRCh38	MIMAT0000158	UGAGAACUGAAUUCCAUGGGUU
**mmu-mir-21a-5p**	Chr.17: 59841266 - 59841337 [+] on Build GRCh38	MIMAT0000530	UAGCUUAUCAGACUGAUGUUGA
**mmu-mir-182-5p**	Chr.6: 30165918 - 30165992 [-] on Build GRCm38	MIMAT0000211	UUUGGCAAUGGUAGAACUCACACCG
**mmu-mir-183-5p**	Chr.7: 129774905 - 129775014 [-] on Build GRCh38	MIMAT0000212	UAUGGCACUGGUAGAAUUCACU
**mmu-mir-451a**	Chr.17: 28861369 - 28861440 [-] on Build GRCh38	MIMAT0001632	AAACCGUUACCAUUACUGAGUU

Accession number available at http://mirbase.org/.

Table S2. Nucleotide sequences of the primers used for RT-PCR of genes.

Gene	Accession Number (#)	Primer sequences (5′ to 3′)
**Traf6**	NM001303273	F: AACGTCCTTTCCAGAAGTGC<br>R: GAATGTGCAAGGGATTGGAG
**Stat3**	NM011486	F: TGTTGGAGCAGCATCTTCAG<br>R: GCATGTCTCCTTGGCTCTTG
**Camk2a**	NM001286809	F: ATTTCCCATCACCAGAATGG<br>R: ATGGCTCCCTTCAGTTTCCT
**Cyclophilin A**	NM008907	F: GTCTCCTTCGAGCTGTTTGC<br>R: GATGCCAGGACCTGTATGCT

Accession number available at http://mirbase.org/.

Table S3. Targets potentially regulated by miR-146a-5p and miR-21a-5p using the Mirnet platform.

ID	Accession	Target	TargetID	Experiment	Literature	Tissue
mmu-mir-146a-5p	MIMAT0000158	Relb	19698	Flow//GFP reporter assay//Immunofluorescence//Luciferase assay//qRT-PCR	22545247	Unspecified
mmu-mir-146a-5p	MIMAT0000158	Tnni1	21952	HITS-CLIP	23142080	Unspecified
mmu-mir-146a-5p	MIMAT0000158	IRAK1	16179	qRT-PCR	22851573	Unspecified
mmu-mir-146a-5p	MIMAT0000158	IRAK1	16179	Luciferase assay//Western blot//qRT-PCR	24358114	Unspecified
mmu-mir-146a-5p	MIMAT0000158	RNF11	30049	Luciferase assay	23345523	Unspecified
mmu-mir-146a-5p	MIMAT0000158	Ndor1	78797	HITS-CLIP	23142080	Unspecified
mmu-mir-146a-5p	MIMAT0000158	Rsad2	56771	HITS-CLIP	23142080	Unspecified
mmu-mir-146a-5p	MIMAT0000158	Sgk3	266632	HITS-CLIP	23142080	Unspecified
mmu-mir-146a-5p	MIMAT0000158	Gpr157	269604	HITS-CLIP	21258322	Unspecified
mmu-mir-146a-5p	MIMAT0000158	Camk2a	12322	HITS-CLIP	19536157	Unspecified
mmu-mir-146a-5p	MIMAT0000158	Camk2a	12322	HITS-CLIP	21258322	Unspecified
mmu-mir-146a-5p	MIMAT0000158	Camk2a	12322	HITS-CLIP	23597149	Unspecified
mmu-mir-146a-5p	MIMAT0000158	Cd93	17064	HITS-CLIP	25083871	Unspecified
mmu-mir-146a-5p	MIMAT0000158	Notch1	18128	Luciferase assay	21730286	Unspecified
mmu-mir-146a-5p	MIMAT0000158	Ifng	15978	Western blot	18791161	Unspecified
mmu-mir-146a-5p	MIMAT0000158	Stat1	20846	Immunoblot//Luciferase assay//qRT-PCR//Western blot	20850013	Unspecified
mmu-mir-146a-5p	MIMAT0000158	Mllt3	70122	HITS-CLIP	25083871	Unspecified
mmu-mir-146a-5p	MIMAT0000158	Irak1	16179	Western blot	18791161	Unspecified
mmu-mir-146a-5p	MIMAT0000158	Irak1	16179	Western blot	23252865	Unspecified
mmu-mir-146a-5p	MIMAT0000158	Irak1	16179	Immunoblot//Luciferase assay//qRT-PCR//Western blot	20850013	Unspecified
mmu-mir-146a-5p	MIMAT0000158	Irak1	16179	Western blot	19596990	Unspecified
mmu-mir-146a-5p	MIMAT0000158	Nos2	18126	Western blot	18791161	Unspecified
mmu-mir-146a-5p	MIMAT0000158	Irak2	108960	Luciferase assay//Western blot	19596990	Unspecified
mmu-mir-146a-5p	MIMAT0000158	Nrp2	18187	HITS-CLIP	25083871	Unspecified
mmu-mir-146a-5p	MIMAT0000158	Map1b	17755	Luciferase assay//Western blot	23699512	Unspecified
mmu-mir-146a-5p	MIMAT0000158	Med1	19014	Luciferase assay	23221399	Unspecified
mmu-mir-146a-5p	MIMAT0000158	Traf6	22034	Immunoblot//Luciferase assay//qRT-PCR//Western blot	20850013	Unspecified
mmu-mir-146a-5p	MIMAT0000158	Traf6	22034	qRT-PCR	22851573	Unspecified
mmu-mir-146a-5p	MIMAT0000158	Slc47a1	67473	HITS-CLIP	25083871	Unspecified
mmu-mir-146a-5p	MIMAT0000158	Traf6	22034	Luciferase assay//Western blot//qRT-PCR	24358114	Unspecified
mmu-mir-146a-5p	MIMAT0000158	Slc47a1	67473	HITS-CLIP	21258322	Unspecified
mmu-mir-146a-5p	MIMAT0000158	Traf6	22034	Western blot	19596990	Unspecified
mmu-mir-146a-5p	MIMAT0000158	Traf6	22034	Western blot	23252865	Unspecified
mmu-mir-21a-5p	MIMAT0000530	Gt(ROSA)26Sor	NA	Immunohistochemistry//Luciferase assay//qRT-PCR//Southern blot	23238710	Unspecified
mmu-mir-21a-5p	MIMAT0000530	Rpp40	208366	HITS-CLIP	21258322	Unspecified
mmu-mir-21a-5p	MIMAT0000530	Eif4e3	72325	Luciferase assay//qRT-PCR	24891504	Unspecified

mmu-mir-21a-5p	MIMAT0000530	Tnfaip8l2	69774	ChIP-seq//Flow//Luciferase assay//qRT-PCR//Western blot	24577093	Unspecified
mmu-mir-21a-5p	MIMAT0000530	Pdcd10	56399	Luciferase assay//qRT-PCR	24891504	Unspecified
mmu-mir-21a-5p	MIMAT0000530	YOD1	234865	qRT-PCR//Luciferase assay//Western blot	24722419	Unspecified
mmu-mir-21a-5p	MIMAT0000530	AK010878	100233175	HITS-CLIP	21258322	Unspecified
mmu-mir-21a-5p	MIMAT0000530	Gid4	66771	HITS-CLIP	23142080	Unspecified
mmu-mir-21a-5p	MIMAT0000530	Mmp9	17395	Immunohistochemistry//In situ hybridization//qRT-PCR//Western blot	23443810	Unspecified
mmu-mir-21a-5p	MIMAT0000530	Cyfip1	20430	HITS-CLIP	25083871	Unspecified
mmu-mir-21a-5p	MIMAT0000530	E2f2	242705	HITS-CLIP	23142080	Unspecified
mmu-mir-21a-5p	MIMAT0000530	Kcnk6	52150	HITS-CLIP	25083871	Unspecified
mmu-mir-21a-5p	MIMAT0000530	Pias3	229615	Western blot	22517757	Unspecified
mmu-mir-21a-5p	MIMAT0000530	Spry1	24063	Luciferase assay//Western blot	20216554	Unspecified
mmu-mir-21a-5p	MIMAT0000530	Fasl	14103	Luciferase assay//Western blot//Northern blot//Reporter assay//Western blot//Other	20404348	Unspecified
mmu-mir-21a-5p	MIMAT0000530	Map3k1	26401	HITS-CLIP	25083871	Unspecified
mmu-mir-21a-5p	MIMAT0000530	Pten	19211	Reporter assay	20167875	Unspecified
mmu-mir-21a-5p	MIMAT0000530	Tgfbr3	21814	Luciferase assay//qRT-PCR//Western blot	22960625	Unspecified
mmu-mir-21a-5p	MIMAT0000530	Moap1	64113	HITS-CLIP	21258322	Unspecified
mmu-mir-21a-5p	MIMAT0000530	Rmnd5a	68477	HITS-CLIP	23597149	Unspecified
mmu-mir-21a-5p	MIMAT0000530	Pten	19211	Luciferase assay//Western blot	19147652	Unspecified
mmu-mir-21a-5p	MIMAT0000530	Pdcd4	18569	Western blot	20404348	Unspecified
mmu-mir-21a-5p	MIMAT0000530	Yy1	22632	Luciferase assay//qRT-PCR	24891504	Unspecified
mmu-mir-21a-5p	MIMAT0000530	Peli1	67245	Luciferase assay//Reporter assay//qRT-PCR//Other	20167875	Unspecified
mmu-mir-21a-5p	MIMAT0000530	Spry1	24063	Western blot	18508928	Unspecified
mmu-mir-21a-5p	MIMAT0000530	Spry1	24063	Luciferase assay//Western blot	19043405	Unspecified
mmu-mir-21a-5p	MIMAT0000530	Reck	53614	Luciferase assay	18556655	Unspecified
mmu-mir-21a-5p	MIMAT0000530	Tgfbi	21810	ELISA//FACS//Luciferase assay//qRT-PCR//Western blot	21494432	Unspecified
mmu-mir-21a-5p	MIMAT0000530	Reck	53614	Western blot	18508928	Unspecified
mmu-mir-21a-5p	MIMAT0000530	Reck	53614	qRT-PCR	21295561	Unspecified
mmu-mir-21a-5p	MIMAT0000530	Reck	53614	Western blot	18372920	Unspecified
mmu-mir-21a-5p	MIMAT0000530	Pdcd4	18569	qRT-PCR	21295561	Unspecified
mmu-mir-21a-5p	MIMAT0000530	Timp3	21859	qRT-PCR	23797704	Unspecified
mmu-mir-21a-5p	MIMAT0000530	Pdcd4	18569	ELISA//Luciferase assay//QRTPCR//Western blot	25000828	Unspecified
mmu-mir-21a-5p	MIMAT0000530	Spry2	24064	Luciferase assay//Western blot//Reporter assay//Western blot//Other	18508928	Unspecified
mmu-mir-21a-5p	MIMAT0000530	Spry3	236576	Western blot	19147652	Unspecified
mmu-mir-21a-5p	MIMAT0000530	Spry3	236576	Western blot	18508928	Unspecified
mmu-mir-21a-5p	MIMAT0000530	Spry4	24066	qRT-PCR	19043405	Unspecified
mmu-mir-21a-5p	MIMAT0000530	Pdcd4	18569	Luciferase assay//Western blot	18372920	Unspecified
mmu-mir-21a-5p	MIMAT0000530	Pdcd4	18569	qRT-PCR	21273303	Unspecified
mmu-mir-21a-5p	MIMAT0000530	Tns1	21961	HITS-CLIP	23142080	Unspecified
mmu-mir-21a-5p	MIMAT0000530	Smad7	17131	Luciferase assay//Northern blot//QRTPCR//Immunoprecipitation	23784029	Unspecified

NS: No specified

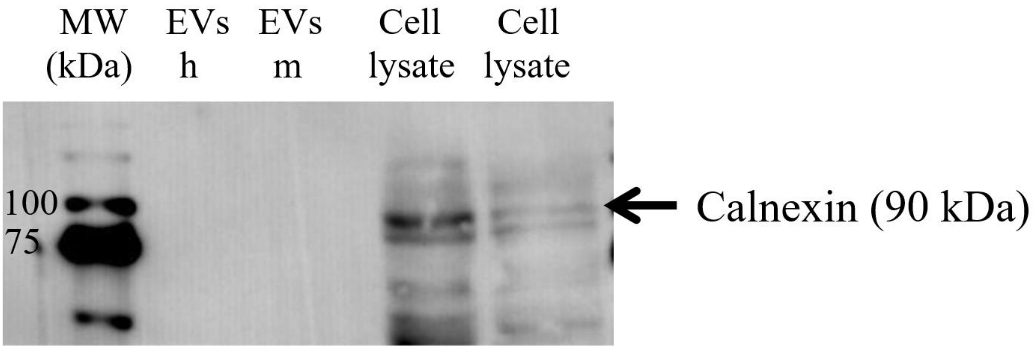

Figure S1. Immunoblot analysis of the calnexin levels present in the EVs from human (h) and murine (m) plasma, and in cellular plasma lysates. Whereas the sample of cellular lysate was use as positive control of the calnexin expression, the absence of the calnexin expression in the EVs samples confirmed the absence of cytosolic protein contamination.

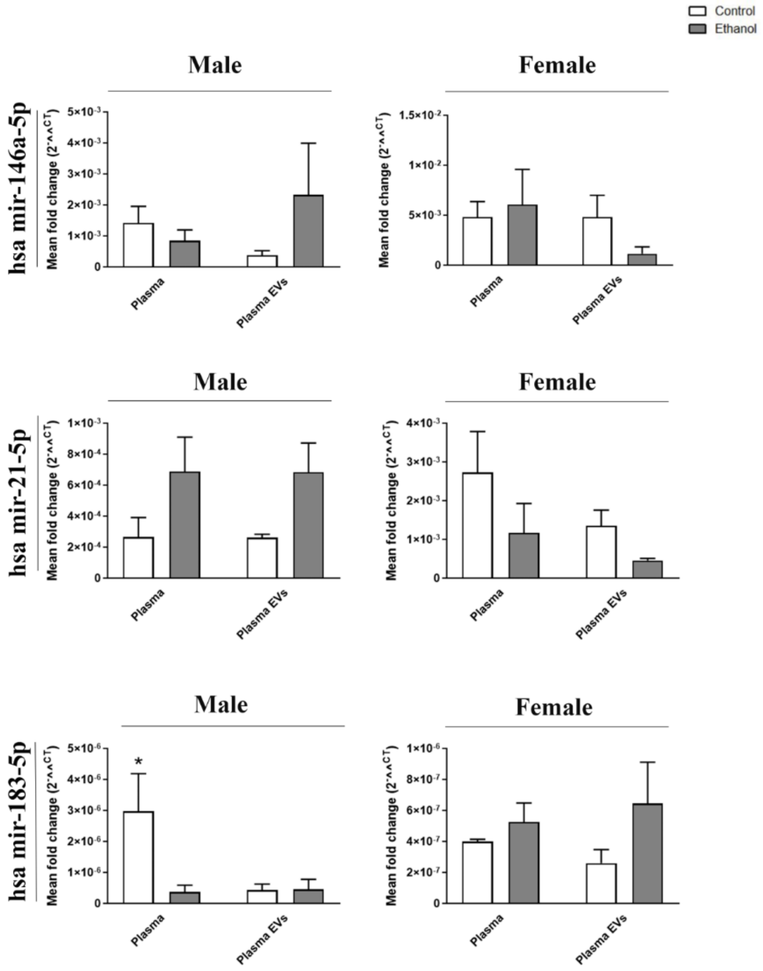

Figure S2. Graphs represent the mir-146a-5p, mir-21-5p and mir-183-5p expression in the plasma and plasma EVs of the human adolescent females and males after acute ethanol intoxication and the data of the corresponding healthy control individuals. Data represent mean±SEM, $n = 6$ independent experiments. * $p < 0.05$ compared to their respective healthy control individuals, according to the two-way ANOVA followed by Bonferroni's post hoc test.

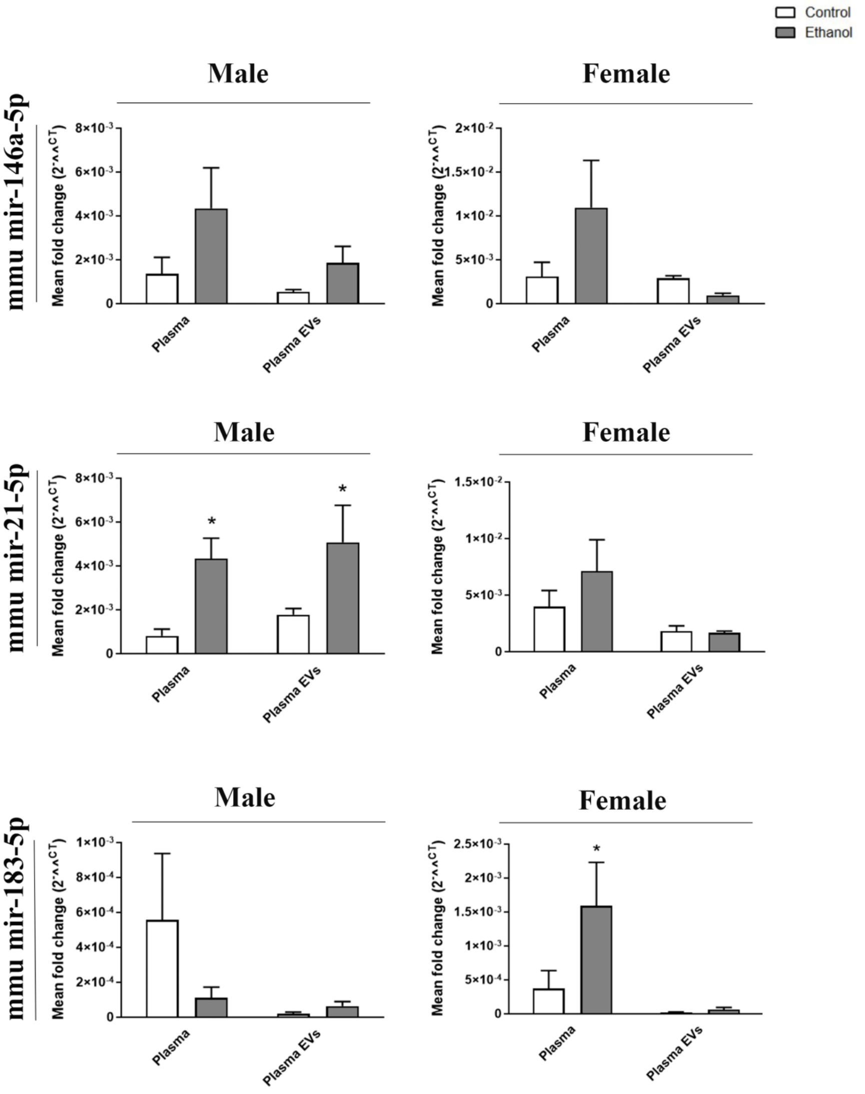

Figure S3. Graphs represent the mir-146a-5p, mir-21-5p and mir-183-5p expression in plasma and plasma EVs after the ethanol or saline treatment in the adolescent female and male WT mice (PND44). Data represent mean±SEM, n=6 independent experiments. * $p < 0.05$ compared to their respective control counterparts, according to the two-way ANOVA followed by Bonferroni's post hoc test.

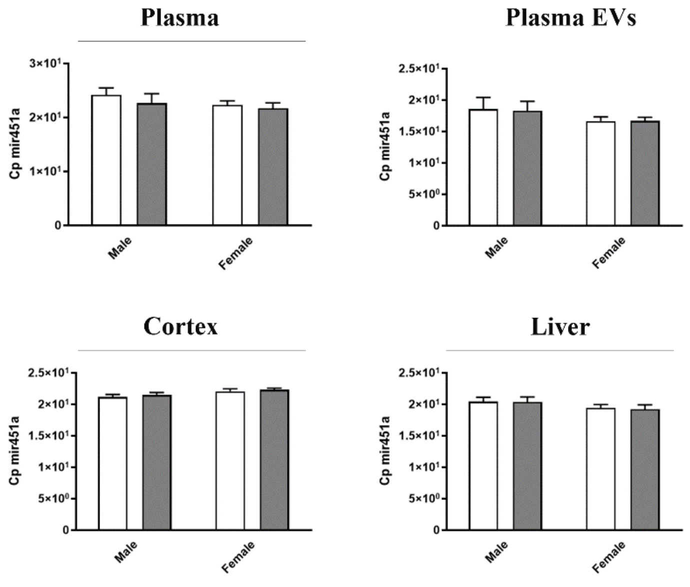

Figure S4. Mir-451a-5p selected as an internal control for miRNAs expression analysis in WT mice.

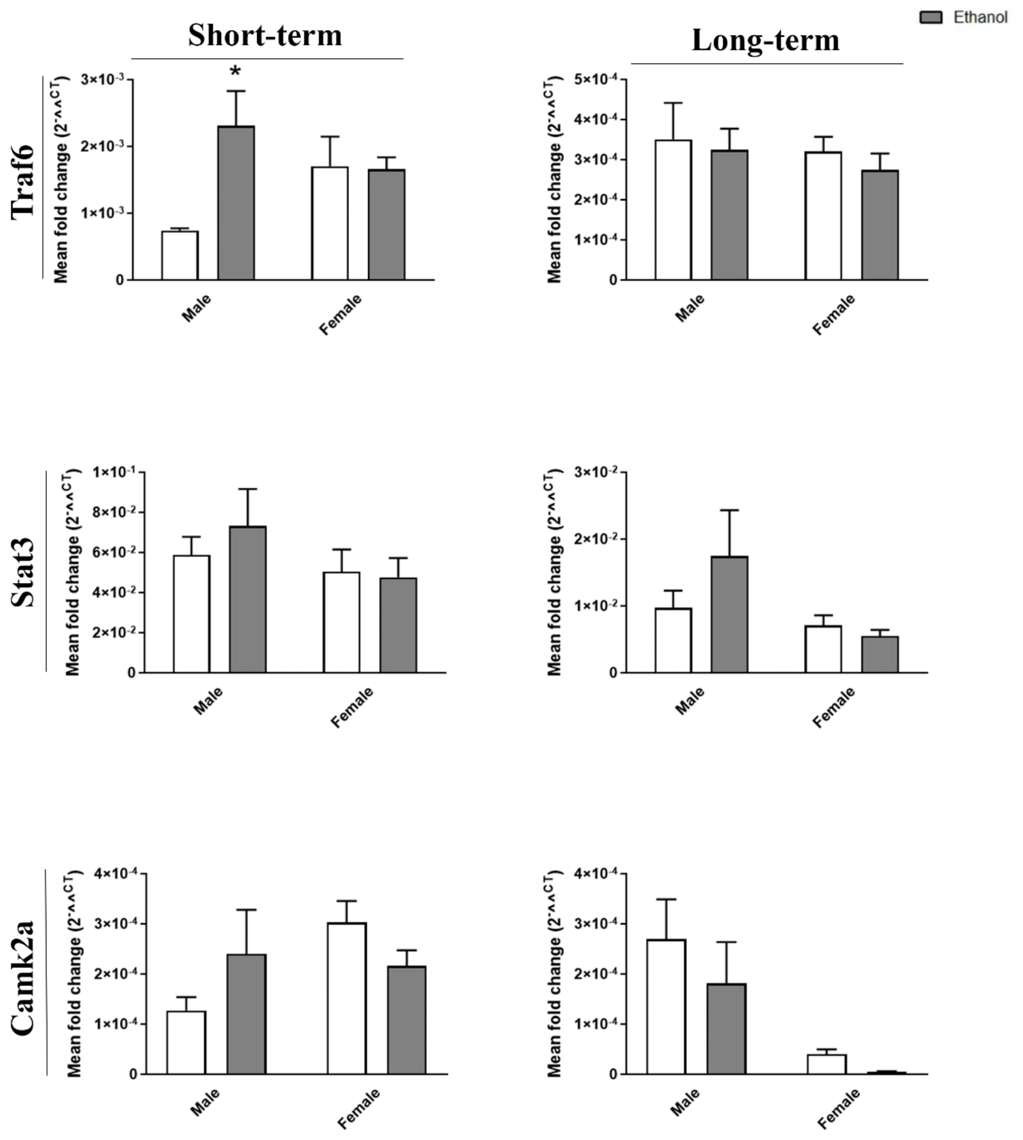

Figure S5. Effects of ethanol on the target gene profile of mir-146a-5p and mir-21-5p in the livers of adolescent WT mice. Graphs represent the expression of Traf6, Stat3 and Camk2a after a 24-hour and 2-week withdrawal period of ethanol or saline treatment in the female and male adolescent WT mice (PND44 and PND58). Data represent mean±SEM, n=6 independent experiments. * $p < 0.05$ compared to their respective control counterparts, according to the two-way ANOVA followed by Bonferroni's *post hoc* test.
